# Source–Detector Spectral Pairing-Related Inaccuracies in Pulse Oximetry: Evaluation of the Wavelength Shift

**DOI:** 10.3390/s20113302

**Published:** 2020-06-10

**Authors:** Olivier Tsiakaka, Benoit Gosselin, Sylvain Feruglio

**Affiliations:** 1 Department of Computer and Electrical Engineering, Université Laval, 1065 Avenue de la Médecine, Quebec, QC G1V 0A6, Canada; benoit.gosselin@gel.ulaval.ca; 2 CERVO Research Center, Quebec, QC G1J 2G3, Canada; 3 Sorbonne Université, CNRS, LIP6, F-75005 Paris, France

**Keywords:** pulse oximetry, wearable sensors, spectrum distribution, wavelength shift, sensor shift, calibration, Beer–Lambert Law, LED

## Abstract

Pulse oximetry enables oxygen saturation estimation ($S_{p}O_{2}$) non-invasively in real time with few components and modest processing power. With the advent of affordable development kits dedicated to the monitoring of biosignals, capabilities once reserved to hospitals and high-end research laboratories are becoming accessible for rapid prototyping. While one may think that medical-grade equipment differs greatly in quality, surprisingly, we found that the performance requirements are not widely different from available consumer-grade components, especially regarding the photodetection module in pulse oximetry. This study investigates how the use of candidate light sources and photodetectors for the development of a custom $S_{p}O_{2}$ monitoring system can lead to inaccuracies when using the standard computational model for oxygen saturation without calibration. Following the optical characterization of selected light sources, we compare the extracted parameters to the key features in their respective datasheet. We then quantify the wavelength shift caused by spectral pairing of light sources in association with photodetectors. Finally, using the widely used approximation, we report the resulting absolute error in $S_{p}O_{2}$ estimation and show that it can lead up to 8% of the critical 90–100% saturation window.

## 1. Introduction

Oxygen saturation measured in blood ($S_{a}O_{2}$) has been assessed non-invasively by pulse oximetry for decades. Pulse oximetry is a relatively easy technique that acquires photoplethysmographs (PPG), which are important bio-signals for physiological assessment. This technology, employed in intensive care units as well as in ambulatory procedures, enables the monitoring of the respiratory condition of the patients and the evaluation of the state of their cardiovascular systems over time. Among its advantages, this technique requires few low-cost components and can give results using a minimal setup [1]. Its ease of use and the derived related patient data (respiratory rate, blood pressure, arterial stiffness, etc.) open the way for advanced applications regarding neurovascular coupling [2,3,4], thus making pulse oximetry an essential tool in hospital routine for rapid patient diagnosis. From basic bedside monitoring in everyday care, its use has extended to diverse applications such as measuring physical exercise performance [5,6,7], oxygen supply at high altitude [8,9], and quality of sleep [10,11]. Capabilities once reserved to high-end facilities are currently becoming widely accessible through dedicated, low-cost, wearable biomedical sensor development kits to replicate the same systems that are currently used in hospitals [12,13,14,15,16,17]. However, there is a a potential for inaccuracy in its primary application for oxygen concentration quantification.

The use of pulse oximetry has long been viewed with some suspicion regarding potential false readings. Indeed, biological sources of inaccuracy such as skin pigments as well as intravenous dyes and non-functional compounds of hemoglobin (the dyshemoglobins) are known to hinder the oxygen saturation measurement optically, leading to falsely low or high readings [1,18]. Several attempts to overcome this limitation have been investigated, but with no means of reducing the influence of such light absorbents in typical dual wavelength pulse oximetry, designers have to include a certain error margin in their systems and follow performance guidelines according to the standard in place [19].

The generally accepted discrepancy claimed by manufacturers of pulse oximeters, between the invasive measurement involving blood sampling and $S_{p}O_{2}$, is typically contained within an error margin of 4%, when healthy values of $S_{p}O_{2}$ range between 96% and 100%. It is admitted that this level of accuracy is sufficient for regular applications (e.g., respiratory patients with a significant decline in oxygenation) [20]. However, the notable inaccuracy prevents its utilization in advanced research applications as long as the calibration process is performed according to the empirical method (i.e., blood measurement on healthy, young volunteers) [1,21] on typical monitoring sites (finger, ear lobes, tongue). Thus, this 4% margin raises concerns when this technique is applied to fine monitoring or to investigate unusual locations such as the central nervous system [22,23,24,25] for example, mainly because it relies on device-specific characteristics, including the optoelectronic parts and the optical and geometrical properties of the tissues [26,27]. Moreover, the incorrect use of this empirical computational model is not device-specific, therefore inducing additional inaccuracies in the estimation of oxygen saturation. Although sensor testers can be used to validate manufactured systems’ performances [28], we did not find any device-specific methods in the literature dedicated to wavelength shift prediction in the conception phase for oxygen saturation error reduction.

In this paper, we aim to raise awareness among designers that do not have access to calibration data to guide the choice of optoelectrical components in pulse oximetry systems and to shine light on the implications regarding the computational accuracy of $S_{p}O_{2}$. To this end, we address the discrepancies between the Beer–Lambert equation and the handy linear approximation with regards to the real spectrum of light sources, far from the ideal narrow spectral width, and different photodetectors with non-uniform responses. Moreover, in order to assist future designs, a simple evaluation of the disagreement between the previous calculations is produced. Using few parameters available directly in the datasheet of the components, the designer is guided in the choice of components to develop a custom pulse oximeter with improved accuracy and performance. The datasets and the custom scripts used to analyze the models during this study are made publicly available in a dedicated Gitlab repository: https://gitlab.com/otsiakaka/wavelength-shift.

This paper first introduces the theoretical background regarding pulse oximetry and the technological choices. In the next section, we show the experimental setup utilized for this study, and we explain the method used to produce the subsequent results before concluding.

## 2. Theoretical Background

This section introduces the concept of operation for the non-invasive estimation of oxygen concentration in peripheral blood using pulse oximetry. The underlying equations behind the estimation are presented with their typical implementation in systems. The corresponding spectral hypotheses supporting these equations are explained in the second part, and the link with the selection of optoelectrical components is established.

### 2.1. Pulse Oximetry

Pulse oximetry aims at quantifying the overall oxygenation of the monitored subject by measuring the peripheral oxygen saturation ($S_{p}O_{2}$) non-invasively. As such, this method tries to approximate the arterial oxygen saturation ($S_{a}O_{2}$), which is the ratio of oxygenated hemoglobin (oxyhemoglobin) concentration [$HbO_{2}$] to the total hemoglobin concentration in the blood $\left[Hb_{total}\right]$ as follows:

(1) $$S_{a}O_{2}=100*\frac{\left[HbO_{2}\right]}{\left[Hb_{total}\right]}$$

Pulse oximetry is one of the optical spectroscopy techniques which attempts to quantify the chromophores of the blood based on their distinct spectral signature regarding optical absorption. By using optoelectronic components to cast a light flux onto tissues and detecting the transmitted (or reflected) light, an oximeter is capable of estimating the $HbO_{2}$ concentration in the capillaries relative to the rest of the compounds of hemoglobin. The Beer–Lambert Law (BLL) allows the quantification of any chromophore in a media—in the present case, hemoglobins in the tissues—-by relating at a certain wavelength λ the absorbance of the media A(λ), or its corresponding transmittance T(λ), with the incident light on the tissues $I_{0}\left(\lambda \right)$ and the measured output intensity I(λ), as shown in Equation (2):

(2) $$A\left(\lambda \right)=-ln\left(T(\lambda )\right)=-ln\frac{I(\lambda )}{I_{0}\left(\lambda \right)}=\varepsilon_{X}\left(\lambda \right)\left[X\right]d\left(\lambda \right)$$

where $\varepsilon_{X}\left(\lambda \right)$ is the molar extinction coefficient of the chromophore *X*, [X] its concentration in the media and d(λ) the optical path length. Applied to pulse oximetry, the variation of attenuation of the light in time is considered mainly to be due to the blood. Thus, all the compounds of hemoglobin are quantified to yield an accurate estimation of blood oxygenation. However, most pulse oximetry systems only consider oxyhemoglobin and deoxygenated hemoglobin (deoxyhemoglobin, Hb), as shown in Equation (1). The BLL is then extended as follows:

(3) $$A\left(\lambda \right)=\left(\varepsilon_{HbO_{2}}\left(\lambda \right)\left[HbO_{2}\right]+\varepsilon_{Hb}\left(\lambda \right)\left[Hb\right]\right)d\left(\lambda \right)$$

**Figure 1:**
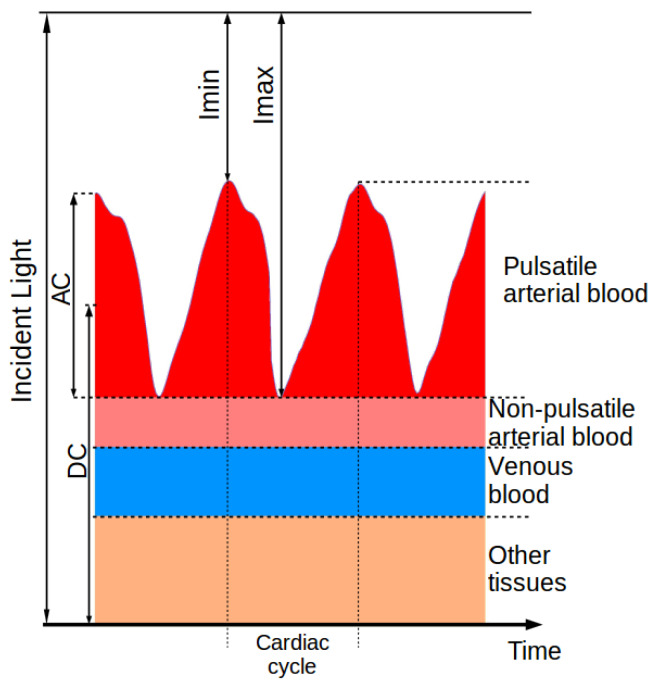
The photoplethysmogram (PPG) signal exhibits a large constant attenuation (DC) and a variable portion (AC) due to the heart rate modulation [22].

The pulsatile arterial blood and other present tissues absorb and modulate the incident light passing through them. This phenomenon forms the photoplethysmogram (PPG) signal, illustrated in Figure 1. The variable portion (AC) of the signal is due to the light that is absorbed by the pulsatile blood (heart rate modulation). A large constant (DC) component is superimposed on it, originating from the light absorbed by soft tissues (e.g., bone, derma, fat, etc.), which is almost invariant in time compared to the period of the heart rate. The AC to DC ratio, referred to as the perfusion index (PI), has long been used to assess tissue blood flow [29].

In order to discriminate the concentration of hemoglobin, a minimum of two light sources is required to solve this equation for two unknowns (see Equation (3)). In the visible and near infrared (NIR) range, considering the blood as the sole variable in time is useful when relating the attenuation of light in the tissues with the oxygenation. One must use two distinct wavelengths where the molar extinction coefficients are sufficiently different, thus measuring two PPG signals at $\lambda_{1}$ and $\lambda_{2}$ to compute the ratio of PIs at each wavelength, defined by

(4) $$R\left(t\right)=\frac{AC\left(\lambda_{1}\right)\;/\;DC\left(\lambda_{1}\right)}{AC\left(\lambda_{2}\right)\;/\;DC\left(\lambda_{2}\right)}$$

where, as shown in Figure 1, $AC(\lambda_{i})$ and $DC(\lambda_{i})$ are the peak-to-peak component and the mean value of $PPG(\lambda_{i})$ over one cardiac cycle, respectively. This method, referred to as the peak and valley method, is convenient when designing an oximeter as one can easily work with the PPG signal to give an estimate of the $S_{p}O_{2}$ without having to compute the distinct concentration values first. Theory shows that this ratio of ratios R(t) is related with the modulation of absorption in time and the change of hemoglobin concentrations in

(5) $$R\left(t\right)=\frac{dA(\lambda_{1},t)}{dA(\lambda_{2},t)}=\frac{\left(\varepsilon_{HbO_{2}}\left(\lambda_{1}\right)\left[HbO_{2}\right]\left(t\right)+\varepsilon_{Hb}\left(\lambda_{1}\right)\left[Hb\right]\left(t\right)\right)\Delta d\left(\lambda_{1}\right)}{\left(\varepsilon_{HbO_{2}}\left(\lambda_{2}\right)\left[HbO_{2}\right]\left(t\right)+\varepsilon_{Hb}\left(\lambda_{2}\right)\left[Hb\right]\left(t\right)\right)\Delta d\left(\lambda_{2}\right)},$$

provided that the modulated difference of the optical path lengths can be approximated as equal for all the wavelengths; i.e. $\Delta d\left(\lambda_{1}\right)\approx \Delta d\left(\lambda_{2}\right)$. Finally, oxygen saturation can be computed with the following equation, according to the BLL:

(6) $$S_{p}O_{2}\left(t\right)=\frac{\varepsilon_{Hb}\left(\lambda_{1}\right)-\varepsilon_{Hb}\left(\lambda_{2}\right)R\left(t\right)}{\varepsilon_{Hb}\left(\lambda_{1}\right)-\varepsilon_{HbO_{2}}\left(\lambda_{1}\right)+\left[\varepsilon_{HbO_{2}}\left(\lambda_{2}\right)-\varepsilon_{Hb}\left(\lambda_{2}\right)\right]R\left(t\right)}$$

**Figure 2:**
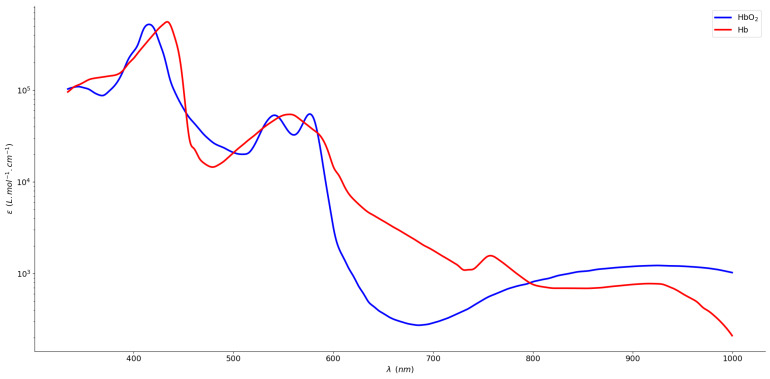
Extinction coefficients spectra of oxyhemoglobin and deoxyhemoglobin in the visible and NIR range, adapted from [32].

In practice, other computational approaches are preferred in place of the absolute quantification of oxygen saturation defined by the previous equation. Following a necessary calibration phase of the system (which conducts to a lookup table), designers may use those hard-coded values in memory to produce the $S_{p}O_{2}$ estimation corresponding to the measured R(t) value [21,30]. However, without access to preliminary measurements on healthy subjects or a suitable reference adapted to the object of study for device-specific calibration, present systems base their estimation on empirical data through curve fitting (i.e., indirect calibration). To facilitate the implementation at low computational cost, the estimation of $S_{a}O_{2}$ uses different degrees of approximation, with the simpler and most used form being the following linearized equation:

(7) $$S_{p}O_{2}\left(t\right)=aR\left(t\right)+b$$

where *a* and *b* are fitting parameters of Equation (6). They are typically extracted from Figure 2. The widely used values for the previously mentioned parameters correspond to extinction coefficients at the typical wavelengths values of 660 nm and 940 nm [1,31], resulting in the following $S_{p}O_{2}$ calculation for a great number of oximetry systems:

(8) $$S_{p}O_{2}\left(t\right)=110-25*R\left(t\right)$$

Note that in the BLL and the definition of the ratio of ratios, several hypotheses must be made to yield Equation (8); mainly, the light sources are presumed to be monochromatic, which means that the illumination spectrum is assumed to be very narrow, and moreover, that the photodetector responsivity is the same across all considered wavelengths.

In the rest of this paper, we first report how the use of the derived values at the typical wavelengths of those parameters induces an error due to the incorrect corresponding value of the molar extinction coefficients $\varepsilon_{X}\left(\lambda \left(i\right)\right)$. Secondly, we show that the distribution spectra shifts the effective peak wavelengths, even for light sources at 660 nm and 940 nm, which can conduct to a significant error of the $S_{p}O_{2}$ estimation that must be taken into account when using photodetectors with non-uniform responses.

### 2.2. Modeled Spectra

In this section, the spectral response of silicon photodetectors and the emission spectra of light-emitting diodes (LED), both typically employed for pulse oximetry, are presented and their impacts are discussed. Both spectra are dependent on temperature, process and supply variations. These unwanted effects are not the topic of this paper, but can be taken into account in the following proposed generic models.

**Figure 3:**
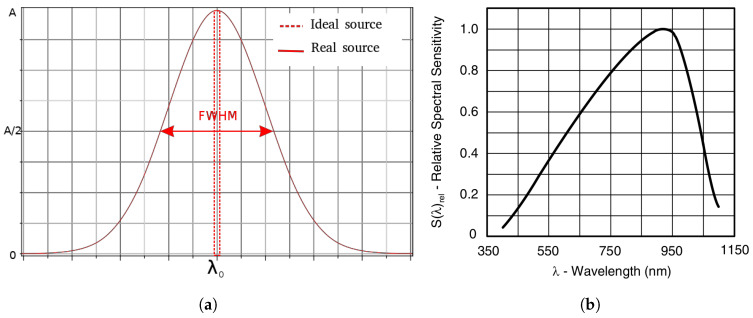
(**a**) Gaussian model typically used for source distribution spectra, compared to the idealistic narrow spectrum (**b**) Relative spectral sensitivity of a typical silicon photodiode [35].

An illustration of a typically used light source in oximetry can be found in Figure 3a, where a Gaussian curve models the actual spectral distribution. In contrast with the infinitely narrow monochromatic spectrum that would resemble that of a tabletop laser, a typical LED has the advantages of being consumer-available, low-cost, chip-scale and power-efficient. For the optical measurement, the drawbacks are visible in the resulting bell shape of the emitted intensity $I_{LED}\left(\lambda \right)$, fitting the maximum value of the curve at $\lambda_{peak}$, and its symmetrically spreading distribution spectrum around that peak according to the full width at half maximum (FWHM), denoted as Δλ, in the following normalized equation:

(9) $$I_{LED}\left(\lambda \right)=e^{-4ln\left(2\right){\left(\frac{\lambda -\lambda_{peak}}{\Delta \lambda_{LED}}\right)}^{2}}$$

Therefore, in contrast to the monochrome hypothesis of the light sources for the use of the BLL resulting in Equation (6), the molar extinction coefficient for one LED is now a function of the wavelengths, thus impacting the oxygenation estimation [33,34].

Moreover, the spectral sensitivity of the photodetector $H_{PD}\left(\lambda \right)$ is not ideally flat. In reality, it is not constant along the wavelengths and exhibits a maximum according to the material characteristics. For the purpose of this paper, and due to the lack of a closer yet simple model, we also modeled this response by a Gaussian curve in

(10) $$H_{PD}\left(\lambda \right)=e^{-4ln\left(2\right){\left(\frac{\lambda -\lambda_{p}}{\Delta \lambda_{PD}}\right)}^{2}}$$

where $\lambda_{p}$ is the wavelength of peak sensitivity and Δλ the range of spectral bandwidth. Figure 3b shows the spectral distribution of the popular BPW34 photodiode used for low-cost oximeters, where the peak sensitivity and the FWHM can be extracted for the model. It is important to keep in mind that the photodetector response will sum all the wavelength contributions in the same PPG. Consequently, the resulting photocurrent generated by the optoelectronic spectral responses affects the ratio defined in Equation (4). In summary, the effective value of the extinction coefficient, denoted as $\varepsilon^{eff}$, is described by the following:

(11) $$\varepsilon_{X}^{eff}\left(\lambda \right)=\sum_{i}\varepsilon_{X}\left(\lambda_{i}\right)I_{LED}\left(\lambda_{i}\right)H_{PD}\left(\lambda_{i}\right)$$

The product of two Gaussian curves is a bell curve of the same type with a resulting FWHM and center wavelength. One can estimate the effective parameters of this curve using the following equation:

(12) $$\Delta \lambda_{eff}\approx \frac{\Delta \lambda_{LED}}{\sqrt{8ln(2)}}$$

The shift from the initial illuminating value δλ can be approximated using the following equation for the resulting effective centre wavelength:

(13) $$\lambda_{eff}\approx \lambda_{LED}+\underset{=\delta \lambda }{\underbrace{\lambda_{PD}{\left(\frac{\Delta \lambda_{LED}}{\Delta \lambda_{PD}}\right)}^{2}}}$$

Note that, in Equation (13), the applicable sign of the shift depends on the value of $\left(\lambda_{LED}-\lambda_{PD}\right)$, as it tends to bring the center wavelengths together.

**Figure 4:**
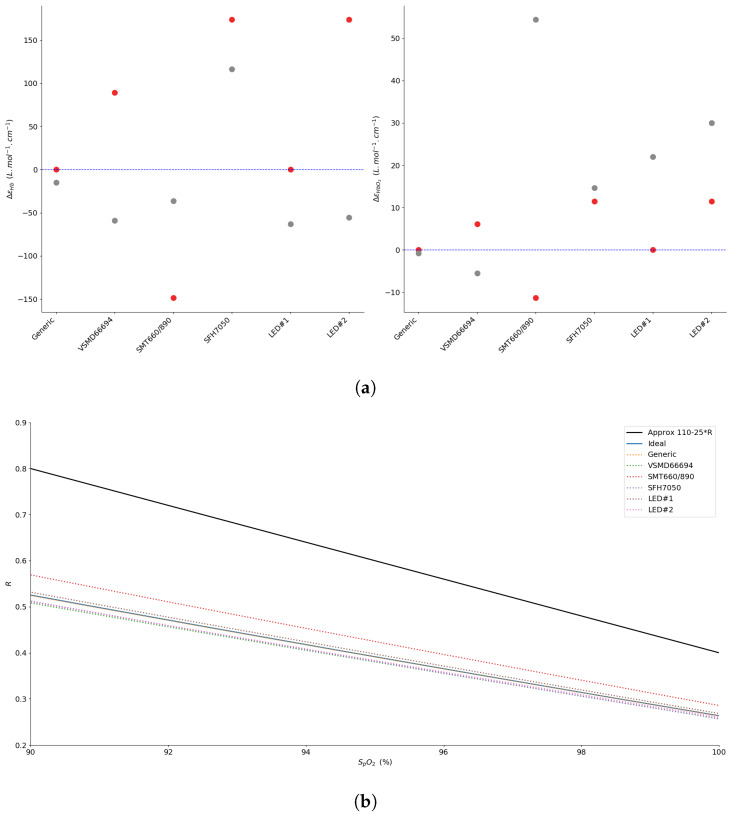
(**a**) Evaluation of the differential shift from the standard values of *ε_Hb_* and *ε_HbO_2__* for different pairs of sources (red dots for the red source and grey dots for the IR source) with the TSL12T photodetector. (**b**) Simulation of the deviation from the linear approximation according to the Beer–Lambert Law (BLL) for different R-IR sources, zoom in the 90–100% region of saturation.

Since evaluating that the error induced by the wavelength shift strictly depends on the extinction coefficients, computing the effective value of $\varepsilon_{X}$ cannot be directly integrated in the $S_{p}O_{2}$ estimation. However, using the optical parameters of the components, we show in Figure 4a the effective values of the shift for $\varepsilon_{Hb}$ and $\varepsilon_{HbO_{2}}$ using the selected light sources of this study. We paired them with regard to oximetry measurements; thus, one is in the red region of the spectrum and one in the NIR. Note that, here, the generic pair refers to a generic red and infrared pair with a peak emission at 660 nm and 940 nm, respectively, and with a spectral width of 20 nm and 50 nm, respectively. As illustrated, in contrast to the ideal case in which the two sources in the pair have a monochromatic distribution (FWHM = 1 nm), all pairs result in a different value for the extinction coefficient of Hb and $HbO_{2}$, thus producing an incorrect estimation of the oxygenation if the linear Equation (8) is used in conjunction in the system. Here, we use the TSL12T photodiode’s extracted characteristics [36], where each pair lends to an absolute maximum shift for $\varepsilon_{Hb}$ of $174\;L\cdot {\mathrm{mol}}^{-1}\cdot {\mathrm{cm}}^{-1}$ when the absolute maximum shift for $\varepsilon_{HbO_{2}}$ is limited at $54\;L\cdot {\mathrm{mol}}^{-1}\cdot {\mathrm{cm}}^{-1}$. This difference in magnitude is explained by the fact that LEDs in the NIR spectrum tend to exhibit a wider wavelength distribution than their counterparts in the visible range, as we present in the optical characterization work shown later in this paper.

In Figure 4b, we evaluate the result of the effective molar extinction coefficients on the estimation of $S_{p}O_{2}$. As illustrated, we can see the difference between the widely used linear approximation and the effective BLL curve for each pair according to the ratio of perfusion indexes. This representation, zoomed in the 90–100% region of saturation, gives an insight into the absolute error produced by pulse oximetry systems when using the linear approximation. Our results show that in this critical saturation area, where an error of 2% in the estimation of the saturation can already lead to an incorrect medical diagnosis [28], the $S_{p}O_{2}$ can be overestimated by the linear approximation by up to 7%.

## 3. Materials and Methods

In the previous section, we have shown how the LED spectra and the photodetector sensitivity, with their Gaussian-like broad peaks, can lead to shifted oxygenation measurements. In this section, we introduce our experimental setup for the collection of real emission spectra from our selected light sources, and we present the key optical parameters extracted.

### 3.1. Experimental Setup

**Figure 5:**
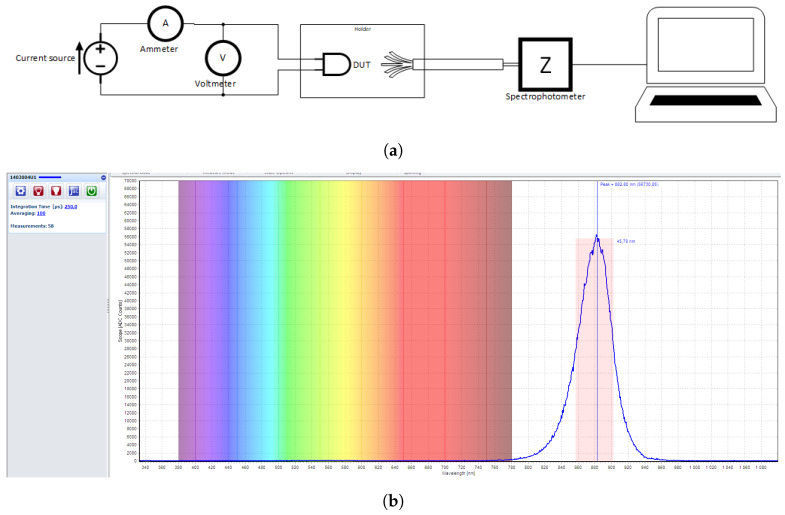
(**a**) Experimental measurement diagram of the setup used for spectra collection, (**b**) Screenshot of an emission spectrum visualized through the software Avasoft with live identification of the full width at half maximum (FWHM) and the peak emission.

The experimental setup is illustrated in Figure 5a. Each device under test (DUT) is placed at the tip of the optical fiber using a customized holder to ensure a precise axial alignment with the core of the optical fiber FC-UVIR1000-2-ME (Avantes, Inc., Apeldoorn, The Netherlands). The light sources are driven with a stable current source, following the conditions in the respective datasheet, if mentioned; if not, the standard forward current $I_{F}=20\;\mathrm{mA}$ is used as the driving parameter value.

The emitted photons are collected by the AvaSpec-ULS2048XL-USB2 spectrophotometer. This equipment has a spectral resolution of 0.6 nm and covers the spectral range from 333 nm to 1100 nm, which suits our purposes for the near infra-red (NIR) range. Figure 5b shows the companion software Avasoft used to configure the acquisition, with an example of an acquired spectrum presented, plotted along with the wavelength with the visible spectrum in the background for reference. Since each source has a different quantum efficiency, we selected a suitable integration time for each one to allow the collection of an appropriate number of photons (arbitrary count unit in the software) to visualize the spectrum with no saturation. The measurements are averaged 100 times to reduce the variability. A dark correction is also applied to remove the influence of eventual light noise. The user is able to visualize in real time the emission spectrum of the device under test (DUT), as well as identifying the peak wavelength and the FWHM of the source.

After visualization, the spectrum can be stored in a comma-separated values file for further computation. For our study, we imported the spectra for manipulation with Spyder (Python 3.7). All spectra, modeled and imported, are aligned on the same wavelength vector and normalized for comparison. We over-sampled the sensitivity spectra of the extinction coefficients and the photodetectors to reach the same resolution as the source spectra.

### 3.2. Selected Components

Table 1 summarizes the optical characteristics of the light sources that we selected for this study. The LEDs characterized in this paper cover the typical range for pulse oximetry, starting from around 620 nm up to 950 nm. One green light source is also included for completeness, as it is part of a triple-wavelength device and could be used to extract the respiratory rate of the subject from the corresponding PPG, thus providing an example of the main artifact of PPG for subsequent removal [37,38]. The parameters mentioned in their respective datasheet are presented to evaluate the impact of the actual spectra relative to the manufacturer’s data and the Gaussian modeling.

In some cases, manufacturers report two specific wavelengths for a light source, $\lambda_{dom}$ and $\lambda_{peak}$, which are the dominant wavelength and the wavelength at peak emission, respectively. In our study, when both are reported, the former is used in the Gaussian model, as it typically corresponds to the highlighted optical key feature for an LED. In the case of the VSDM66694 and the SMT660/890, only the peak emission is reported, so we used this value in our models. In the case of the commercial devices, few values could be found for the parameters of the Gaussian curve; thus, only the measured values could be used in our models.

We compare in this table the manufacturer’s values with our measured parameters on the right-hand side, where the peak emission and the FWHM are extracted with the spectrophotometer companion software. The dominant corresponding wavelength is not computed as its value is subject to interpretation.

The last lines of our table present two commercial double-wavelength light sources used in everyday care routines. Although their optical performances are comparable to our consumer-available components, as we show here, the simulation using their characteristics underlines how the calibration step is a valuable tool for correcting $S_{p}O_{2}$ estimation errors, when available.

**Figure 6:**
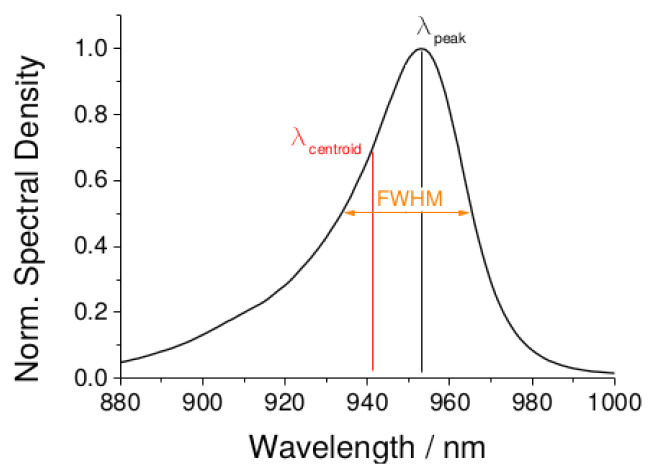
The real model of the distribution spectra of a 940 nm LED source is a skewed Gaussian (left or right leaning), modified from [46].

As mentioned above, some manufacturers provide a better description of the spectrum of the LED with two separate wavelengths. We do not consider multiwavelength sources or secondary peaks here; instead, those two distinct parameters, namely $\lambda_{dom}$ and $\lambda_{peak}$, highlight the skewness of the spectrum distribution. When provided, the dominant wavelength, otherwise noted as the centroid wavelength $\lambda_{centroid}$ or center wavelength $\lambda_{center}$, represents the weighted mean of the spectrum. Thus, an accurate model of the light distribution shifts from a Gaussian bell to a skewed Gaussian. The use of this model has instead been applied to better shape the light source distribution [49]. Figure 6 shows how these parameters fit into the skewed model.

However, the different terms used to mathematically shape the tail and the decay do not fit all cases. Thus, it is still recommended to use a bell curve as best overall representation [50]. Nonetheless, in the absence of a fit for all skewed models, we used the manufacturer’s spectral sensitivity data available for three typical cases of silicon photodiodes with our measured spectra of the LEDs to evaluate the impact on the typical computation of oxygen saturation.

**Figure 7:**
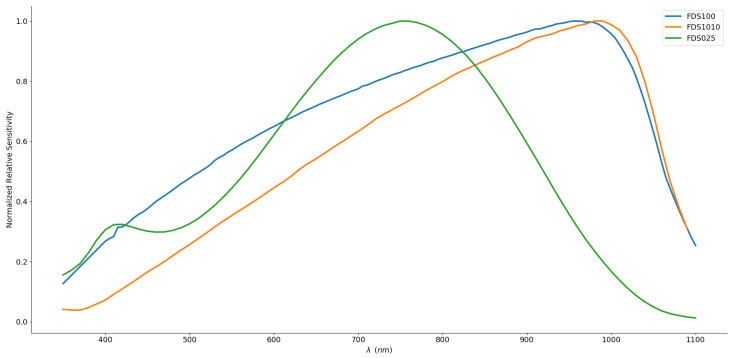
Normalized real sensitivity spectra of three commercial photodetectors (FDS100, FDS1010 and FDS025), adapted from [51].

Figure 7 illustrates real sensitivity spectra extracted from the manufacturers’ measurements. The normalization allows us to appreciate the spectral shape of the selected photodiodes. The curves of the FDS100 and FDS1010 differ greatly from the bell shape. Indeed, the FDS1010 shows a quasi linear ramp along the wavelengths of interest, whereas the FDS025 exhibits a symmetry between the typical chosen wavelengths. This feature has an impact on the sign of the shift presented earlier (see Equation (13)) as the peak sensitivity is located between 660 nm and 940 nm.

Finally, we compute the absolute error in oxygen saturation measurement with pulse oximetry considering the following equation:

(14) $$\Delta SpO_{2}=\left|\frac{BLL-Approx}{BLL}\right|*100\%$$

Here, we compute the error of the approximation (see Equation (8)) in relation to the effective BLL (see Equation (6)) for each selected pair of sources, meaning that the error of the approximation is computed along the *R* axis, taking the effective—i.e., shifted—values of the extinction coefficients.

## 4. Results

We first show in this section the results of our optical characterization for our selected LEDs. The acquired spectra are plotted to illustrate the difference between the ideally narrow distribution and the actual emission. Subsequently, we report the induced errors of these spectra for pulse oximetry measurements, relative to the typical linear approximation.

### 4.1. Measured Spectra

The results presented in this section are computed for each source pair. Dual LEDs and commercial devices are considered as such. One pair, the LHQ974 and SFH4248, is presented as a model for a typical red and typical IR source, regarding their optical specifications, which are common among their respective wavelength range for LED sources.

**Figure 8:**
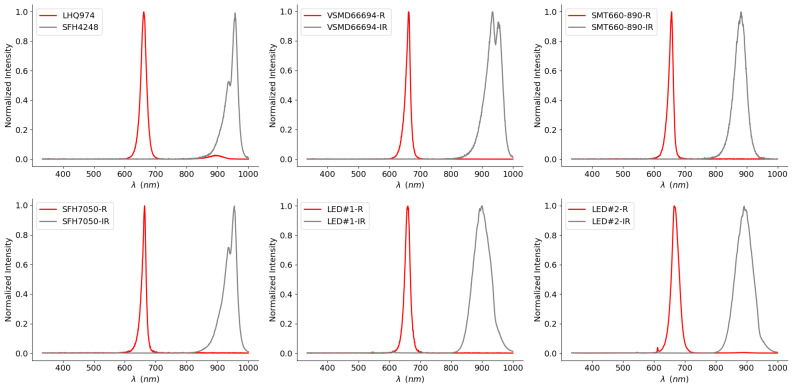
Measured distribution spectra of our light sources by Red–IR pair.

We show in Figure 8 the normalized measured spectra of the light sources selected in this study. Once again, the spectra are paired with consideration to a typical oximetry measurement setup; i.e., one red source is paired with an infrared one. From this point on, the first pair (LHQ974-SFH4248) will be considered as representative of a typical LED pair for oximetry with regard to their spectrum and will be denoted as the typical pair.

As expected, all measured spectra exhibit several differences with the Gaussian model. First, we can observe that the distributions are asymmetrical; indeed, all curves express a skew to the left or the right for all sources. This observation is in agreement with the previously mentioned model (see Figure 6).

Second, almost all of the LEDs have a secondary peak, whether close to the primary peak with a similar intensity (IR LED VSDM66694 at the top center) or less dominant (IR LED SFH4248 at the top left, IR LED SFH7050 at the bottom left). This secondary wavelength can also be distant to the primary peak, as seen with the LHQ974 (at the top left), where the red main emission is accompanied by a small infrared feature. Although observable thanks to the optical characterization, we did not take these stray peaks into consideration in our initial models. Indeed, those are not reported in the datasheet by the manufacturers when the light source is not considered to be multiwavelength (two distinct peaks at equivalent magnitudes in different color ranges). However, in practice, they contribute in the effective value of the extinction coefficient (see Equation (11)).

Even though these deviations from the datasheet specifications (see Table 1) can be attributed to the fabrication process, or the driving voltage-current variations, it appears clear that these non-uniformities call for device-specific calibration.

### 4.2. Related Error in Quantification

**Figure 9:**
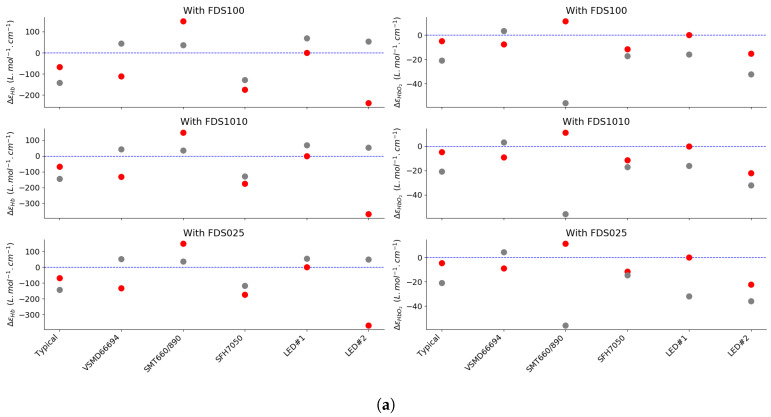

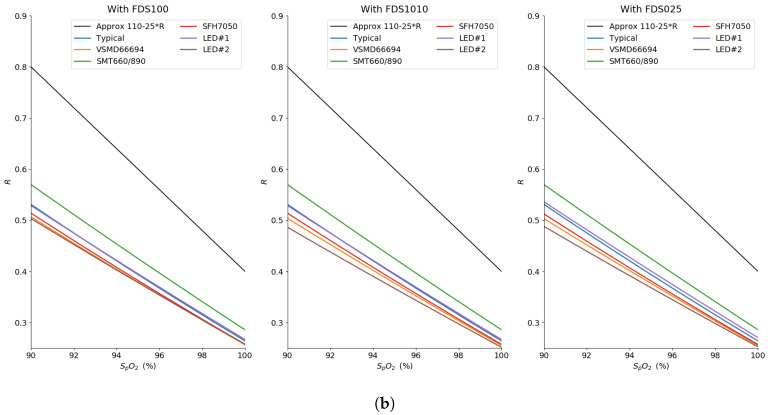
(**a**) Evaluation of the differential shift from the standard values of $\varepsilon_{Hb}$ and $\varepsilon_{HbO_{2}}$ for our selected source pairs (red dots for the red source and grey dots for the IR source) (**b**) Simulation of the deviation from the linear approximation according to the BLL for four our selected R/IR source pairs, zoom in the 90–100% region of oxygen saturation.

The absolute extinction coefficient difference is presented in Figure 9a, where each LED pair is evaluated as an emitter in pulse oximetry, with the aforementioned photodetectors (see Figure 7). As expected in Equation (11), the different spectral sensitivities of the photodiode have an impact on the effective extinction coefficient for each hemoglobin compound. Here also, the absolute maximum shift is seen on $\varepsilon_{Hb}$. However, one can see that the shift is maximal for light sources with a peak distant from the standard 660 nm and 940 nm but also with a larger FWHM, as anticipated by Equation (13).

In Figure 9b, we illustrate the related deviation from the linear model along the *R* axis. In the worst case with the FDS1010 photodetector, this deviation reaches 8% in the critical 90–100% saturation.

**Figure 10:**
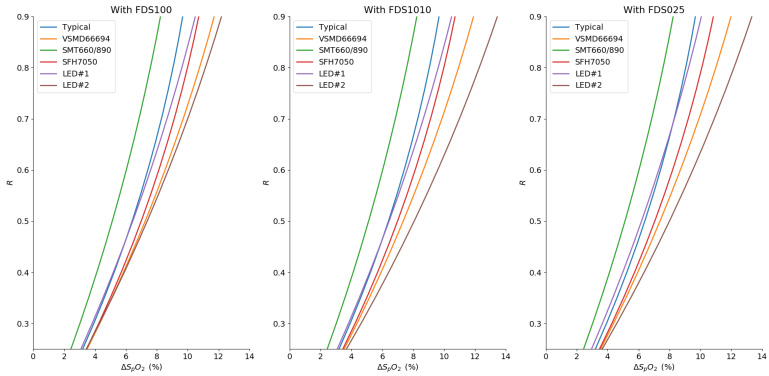
Model prediction of the absolute error of $S_{p}O_{2}$ quantification for selected source pairs with different photodetectors.

Finally, we show the absolute produced error in Figure 10 in the same *R* range, relative to the linear approximation. As displayed, no configuration reaches an absolute error below 2% in the critical window.

## 5. Discussion and Conclusions

Pulse oximetry is a versatile tool to quantify blood oxygen concentration in a non-invasive fashion. It gives access to a vital bio-signal for rapid assessment of the overall condition of the patient as well as local specific hemodynamics. However, in critical conditions, pulse oximetry lacks a general calibration process. With this crucial phase being tissue and device-dependent at the component level, a one-size-fits-all approach is hardly conceivable.

We showed in this paper that, taking into account the effect of the imperfections of the building blocks of a pulse oximeter—namely the light source and the photodetector—one can predict how the measurement will deviate from the absolute quantification provided by the BLL when using linear approximation. In our study, we experimented with several pairs of LED to show how disregarding their optical characteristics can lead to computational inaccuracies. We revealed a $S_{p}O_{2}$ estimation error up to 8% in the 90–100% range of the oxygen saturation. Moreover, through the evaluation of the shift generated by this spectral mismatch and the source–detector pairing, we showed how this pitfall can be avoided at the design phase with the few parameters provided in the datasheets.

Although calibration-free methods are explored, their implementation still remains outside of a reasonable error margin or relies on complex setups. Furthermore, they mostly rely on more expensive light sources such as Vertical-Cavity Surface-Emitting Lasers (VCSELs) for a spectral emission closer to the ideal model [34,52,53]. Despite offering a better optical power to the driving current rate than typical LEDs, these devices require specific care regarding local heating, thus hindering their use in wearables.

In any case, pulse oximetry remains incomplete as long as dyshemoglobin concentrations are not quantified. The shift toward co-oximetry, also named Multi-Wavelength pulse oximetry [1,54], addresses this limitation and improves overall accuracy. However, an interesting additional alternative could be its association with specific multi-layer Si photodetectors, which are well-known to yield a better spectral discrimination and signal-to-noise ratio than usual photodiodes, without real additional manufacturing costs thanks to their fabrication with standard Complementary Metal-Oxide Semiconductor (CMOS) technologies [55,56,57].

Future work may also include a more accurate representation of LED emission distribution to better model the complexity of the spectrum and its variations [50,58]. The photodetector’s sensitivity may also incorporate more complexity, starting with photo-response non-uniformity.

Finally, even though this paper focused on the linear approximation of $S_{p}O_{2}$ estimation, which is widely used due to its simplicity, our considerations regarding emission and reception spectra remain equally valid for more complex approximation models. By placing our attention to wavelength shift, we hope that future designs in critical monitoring systems will take special care in mitigating accuracy issues due to component pairing.

## Figures and Tables

**Table 1:** Selected suitable LEDs for pulse oximetry. All LEDs were tested at room temperature (25 °C), under the same conditions mentioned in their respective datasheet to compare the spectral measurements (i.e., if the forward current specified was 20 mA, we set our current source in the setup as If = 20 mA) [39,40,41,42,43,44,45,46,47,48]. Values marked “x” were not reported in the corresponding datasheet.

Reference	Manufacturer	Package	From Datasheet			Measured	
			$\lambda_{\mathrm{dom}}$ (nm)	$\lambda_{\mathrm{peak}}$ (nm)	Δλ (nm)	$\lambda_{\mathrm{peak}}$ (nm)	Δλ (nm)
LHQ974	OSRAM	SMD	643	660	20	657	19
SFH4050	OSRAM	SMD	850	860	30	857	21
SFH4248	OSRAM	SMD	940	950	42	956	35
VSMD66694	Vishay	SMD	x	660 ± 10	20	662	16
			x	940 ± 20	40	934	57
SMT660/890	Marubeni	SMD	x	650 ± 10	20	657	16
			x	890 ± 15	75	883	46
SFH7050	OSRAM	SMD	530 ± 10	525	34	532	33
			655 ± 3	660	17	664	15
			940 ± 10	950	42	954	44
LED #1	“M”	SMD	660	x	x	660	21
			905	x	x	899	68
LED #2	“D”	SMD	x	x	x	664	22
			x	x	x	895	62

## Abbreviations

The following abbreviations are used in this manuscript:

BLL	Beer Lambert Law
CMOS	Complementary Metal-Oxide Semiconductor
DUT	Device Under Test
FWHM	Full Width at Half Maximum
LED	Light-Emitting Diode
NIR	Near Infra-Red
PI	Perfusion Index
PPG	PhotoPlethysmoGram
SMD	Surface Mount Device
VCSEL	Vertical-Cavity Surface-Emitting Laser
